# Safety and feasibility of atrial fibrillation ablation after left atrial appendage closure: A single‐center experience of the left atrial appendage closure first strategy

**DOI:** 10.1002/joa3.13073

**Published:** 2024-05-23

**Authors:** Ryuki Chatani, Shunsuke Kubo, Hiroshi Tasaka, Atsushi Sakata, Mitsuru Yoshino, Takeshi Maruo, Kazushige Kadota

**Affiliations:** ^1^ Department of Cardiovascular Medicine Kurashiki Central Hospital Kurashiki Japan

**Keywords:** antithrombotic regimen, atrial fibrillation, catheter ablation, left atrial appendage closure, peri‐device leak

## Abstract

**Background:**

Patients with atrial fibrillation (AF) who are not suitable for long‐term anticoagulant therapy undergo percutaneous left atrial appendage closure (LAAC). The safety and feasibility of left atrial catheter ablation (CA) procedures after LAAC remain unclear. This study aimed to clarify the feasibility and safety of CA after LAAC, including in the early phase within 180 days.

**Methods:**

Characteristics and clinical outcomes of 46 patients with AF who had undergone both CA and LAAC within 2 years (mean age, 72 years; 29 men) were compared between those who had undergone CA‐first (31 patients) and LAAC‐first (15 patients).

**Results:**

The mean CHA₂DS₂‐VASc and HAS‐BLED scores were 4.8 and 3.3 points, respectively. The LAAC‐first strategy was often used in patients with prior major bleeding and LAA thrombosis or sludge. In the LAAC‐first group, the mean duration between both procedures was 212 days, and all LAAC‐first patients, including seven patients in the early phase, could undergo CA without LAAC device‐related complications; moreover, no cardiovascular adverse events were reported after both procedures (mean periods: 420 days). After CA post‐LAAC, no device‐related adverse events (device‐related thrombosis, new peri‐device leak appearance, peri‐device leak increase, or device dislodgement) were observed, whereas, after LAAC post‐CA, 3 new peri‐device leak appearance events and 1 peri‐device leak increase event were observed, especially patients who underwent LAAC in the early phase post‐CA.

**Conclusion:**

Based on single‐center experience, left atrial CA in the presence of an LAAC device implanted including the early phase was safe and feasible.

## Graphical abstract

Atrial fibrillation ablation in the presence of an LAAC device implanted including the early phase was safe and feasible without LAAC device adverse events. Contrastingly, when performing LAAC after PVI, it may be better to perform in the late phase, considering the possibility of PDL due to LAA ridge edema.

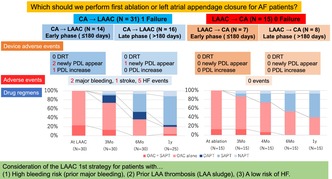

## 1 INTRODUCTION

Catheter ablation (CA) therapy for atrial fibrillation (AF) has been shown to be effective for drug‐resistant symptomatic patients and those with heart failure (HF).^1^, ^2^, ^3^, ^4^ However, the continuation of long‐term oral anticoagulation (OAC) therapy is recommended in patients with a high CHADS₂ score or CHA₂DS₂‐VASc score.^3^, ^4^ Left atrial appendage (LAA) closure (LAAC) has emerged as an alternative to long‐term OAC therapy with similar demonstrated efficacy for stroke prevention.^5^, ^6^, ^7^ Generally, East Asian patients, including Japanese, are small and are at high risk of bleeding.^8^ Therefore, many Japanese patients may be eligible for LAAC. The SALUTE trial in Japan was conducted in 42 Japanese patients at high risk of bleeding and reported the safety of percutaneous LAAC using the conventional WATCHMAN 2.5 system, which was reimbursed in Japan in 2019.^9^ In addition, 2‐year follow‐up outcome data demonstrated that the WATCHMAN LAAC device is an effective and safe alternative nonpharmacological therapy for stroke risk reduction in Japanese NVAF patients who are not optimal candidates for lifelong anticoagulation.^10^ Currently, LAAC has been performed on many Japanese patients, and the safety and effectiveness of the current WATCHMAN FLX device have been demonstrated.^11^, ^12^, ^13^ In addition, 19.3% to 40.5% of patients undergoing LAAC had previously undergone AF ablation in Japan.^10^, ^12^, ^13^ Therefore, Patients with nonvalvular AF may require a combination therapy of CA and LAAC depending on their situation.

Because of the common aspects of transseptal puncture and the need for postprocedural anticoagulation, a combination therapy involving two left atrial interventions may be a valuable and practical approach.^14^, ^15^ The safety of combination therapy has been reported retrospectively^16^, ^17^; moreover, the number of patients being treated with the combination therapy is increasing,^18^ and a prospective study is currently underway.^19^

However, owing to issues such as health insurance, both therapies are often administered separately. Usually, LAAC is performed after CA to avoid LAAC device‐related complications such as device‐related thrombosis (DRT), peri‐device leak (PDL), and device dislodgement, even in the case of combination therapy.^20^, ^21^, ^22^ The feasibility and safety of left atrial CA in the presence of an LAAC device implanted in the late phase (>180 days) have been reported.^23^

However, the feasibility and safety of left atrial CA after LAAC remain unclear, especially in the early phase (≤180 days). Thus, the present study aimed to clarify the feasibility and safety of left atrial CA after LAAC, especially in the early phase. We herein report on 15 patients with AF (including 7 patients in the early phase) who successfully underwent CA after LAAC.

## 2 METHODS

### 2.1 Study design

**FIGURE 1:**
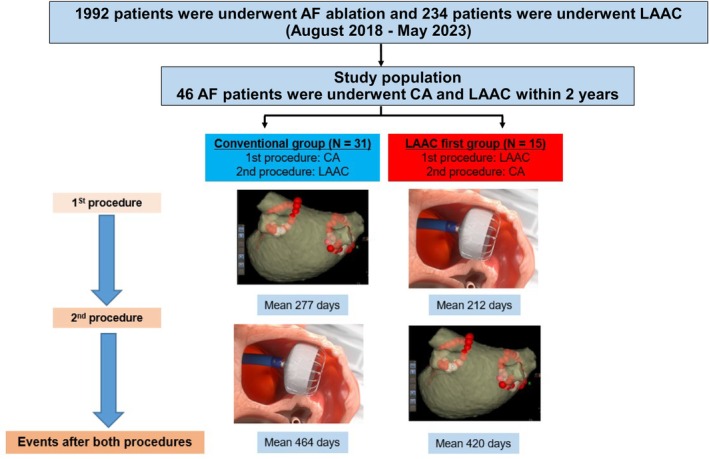
Study flow chart. The mean duration between the CA and LAAC procedures in the CA first group was 277 ± 197 days. The mean duration between the LAAC and CA procedures in the LAAC group was 212 ± 172 days. The mean observation periods for both procedures were 464 ± 277 days in the CA first group and 420 ± 260 days in the LAAC first group. CA, catheter ablation; LAAC, percutaneous left atrial appendage closure.

This investigator‐initiated single‐center interventional study retrospectively analyzed 46 consecutive patients with AF who had undergone CA and LAAC within 2 years between August 2018 and May 2023 at Kurashiki Central Hospital. From August 2018 to May 2023, 1992 patients had undergone AF ablation and 234 patients had undergone LAAC. The analyzed patients were divided into the following two groups based on the procedure they underwent first: CA first group (conventional group that underwent CA first; *n* = 31) and LAAC first group (group that underwent LAAC first; *n* = 15) (Figure 1). The study was approved by the Institutional Review Board of Kurashiki Central Hospital (approval no. 4133), and an opt‐out system was used to obtain patient consent for the use of their clinical data for research purposes. This study was conducted according to the principles of the Declaration of Helsinki.

### 2.2 LAAC procedure

All LAAC procedures were performed under general anesthesia using angiography and transesophageal echocardiography supports. The WATCHMAN 2.5 or WATCHMAN FLX device was used for all patients. All procedures were performed by a certified operator. Intraprocedure transesophageal echocardiography was performed by a certified echocardiologist who had completed a specific training program for the procedure. The target activated clotting time (ACT) during the procedure was 250–300 s, and heparin was administered intravenously. After obtaining femoral vein access, a transseptal puncture was performed using a radiofrequency needle through an SL‐1.0 sheath (St. Jude Medical). Then, we inserted an access sheath in the left superior pulmonary vein and selected LAA using the pigtail catheter. The tip of the access sheath was confirmed in angiography and transesophageal echocardiography, and device delivery catheter was inserted in the sheath. The device size was selected based on the angiography and transesophageal echocardiography measurements of the LAA ostium during the procedure. The device was deployed by unsheathe motion in WATCHMAN 2.5 and by either unsheathe motion or device advancement in WATCHMAN FLX. After device deployment, the PASS criteria (position, anchor, seal, and size) were checked using angiography and transesophageal echocardiography. After confirmation of the PASS criteria, the device was released.

### 2.3 CA procedure

All CA procedures were performed under nongeneral anesthesia, except for one patient with congenital heart disease. All patients were on or resumed OAC before CA. Patients who had undergone CA after LAAC were subjected to screening transesophageal echocardiography or contrast‐enhanced 64‐slice cardiac computed tomography (CT) whenever possible before the planned procedure. The system was chosen at the discretion of the operator. When intracardiac echocardiography (ICE) was performed, the ICE was also used to screen for a DRT of the WATCHMAN device. Pulmonary vein isolation (PVI) was performed in all cases using a cryoballoon or radiofrequency (RF) catheter. Patients requiring additional ablations (e.g., LA substrate ablations, such as low voltage ablation and complex fractionated electrogram, and liner ablations, such as posterior wall isolation, cavo‐tricuspid isthmus isolation, and superior vena cava isolation) underwent treatment at the operator's discretion. This study did not include pulsed‐field ablation. The target ACT value during the procedure was ≥300 s, and heparin was administered intravenously. In cases where the Carto system (Carto 3; Biosense‐Webster, Irvine, CA) was used as the 3D mapping system and CARTOSOUND (Biosense‐Webster) was used as the ICE system, the WATCHMAN device was created in the 3D mapping system and the surface bipolar voltage was observed using an 8‐spline multipolar mapping catheter (Octaray; Biosense‐Webster). Our ridge ablation strategy to achieve PVI without interfering with the LAAC device was as follows: first, we tried to ablate the ridge from the PV side. If PVI was not achieved, we then tried to ablate the ridge from the LAA side near the proximal side to avoid interference with the LAAC device (Figure S1). In this way, PVI was achieved without interfering with the LAAC device.

### 2.4 Clinical follow‐up and endpoints

Follow‐up data were mainly collected through a review of the hospital charts. Additional follow‐up information was collected by contacting patients, relatives, and/or referring physicians via phone and/or mail and asking questions regarding vital status, clinical events, nature of the procedure (invasive), and status of anticoagulation therapy. In this cohort study, final data collection for follow‐up events was performed between September 2022 and March 2024.

The primary endpoint was cardiovascular events after CA and LAAC. The cardiovascular events included ischemic stroke, major bleeding, all bleeding (major and clinically relevant minor bleeding) episodes, HF hospitalization, and all‐cause death. Ischemic stroke was defined as the sudden onset of a focal neurologic deficit in a location consistent with the territory of a major cerebral artery, as confirmed via CT or magnetic resonance imaging. Ischemic stroke was classified as disabling if associated with an increase in the modified Rankin Scale (mRS) score of at least 2 points at discharge.^10^

Major bleeding consisted of fatal bleeding, symptomatic bleeding in a critical area or organ, and bleeding causing a reduction in the hemoglobin level by at least 2 g/dL or leading to transfusion of at least 2 units of whole blood or red cells according to the International Society on Thrombosis and Haemostasis definitions.^24^ Clinically relevant minor bleeding consisted of hospital admission for bleeding, physician‐guided medical or surgical treatment for bleeding, change in antithrombotic therapy, and bleeding that did not meet the criteria for major bleeding. DRT was defined as a thrombus adhering to the device surface as confirmed by cardiac contrast computed tomography (CCT) or transesophageal echocardiography. Residual peri‐device leak was defined by transesophageal echocardiography only, with residual peri‐device leak >5 mm defined as a major leak and ≤5 mm as a minor leak. Complete seal was defined as the complete absence of residual peri‐device leak.^25^

### 2.5 Statistical analysis

Patients were divided into two groups (CA first and LAAC first groups) based on the procedure they underwent first. Categorical variables are presented as numbers and percentages. Continuous variables are presented as means and standard deviations or medians and [25, 75 percentiles] based on their distributions. Categorical variables were compared using chi‐square test. Continuous variables were compared using one‐way analysis of variance or Kruskal–Wallis test based on their distributions. Statistical significance was defined as a *p*‐value of <0.05. SPSS ver. 23 (IBM Corp., Armonk, NY) was used for statistical analyses.

## 3 RESULTS

### 3.1 Patient characteristics at the time of the first procedure

The mean age of the analyzed patients was 72 ± 9 years. Among the analyzed patients, 29 were men and 22 had nonparoxysmal AF. Moreover, the mean CHADS₂, CHA₂DS₂‐VASc, and HAS‐BLED scores were 3.3 ± 1.2, 4.8 ± 1.4, and 3.3 ± 0.8 points, respectively. The bleeding risk and embolism risk were high in the study population. In total, 31 patients (67.4%) were included in the CA first group and 15 patients (36.6%) were included in the LAAC first group.

Patient characteristics, clinical presentations, and procedural characteristics were different in some aspects between the two groups (Table 1). Long‐standing persistent AF and prior major bleeding were more common in the LAAC first group than in the CA first group. Other patient background characteristics were similar between the groups. In the CA first group, the mean duration between the CA and LAAC procedures was 277 ± 197 days, whereas in the LAAC first group, the mean duration between the LAAC and CA procedures was 212 ± 172 days.

**TABLE 1:** Patients characteristics, clinical presentation, and procedural characteristics.

	Conventional group (*N* = 31)	LAAC first group (*N* = 15)	*p*‐value
Patients characteristics			
Age (years)	73.2 ± 10.1	69.2 ± 7.6	0.142
Male sex	19 (61%)	10 (67%)	1.0
Body weight (kg)	59.7 ± 11.3	60.0 ± 12.2	0.925
Body mass index (kg/m^2^)	23.3 ± 3.2	23.1 ± 2.4	0.883
AF type			
Paroxysmal AF	17 (55%)	7 (47%)	0.755
Persistent AF	11 (36%)	3 (20%)	0.331
Longstanding persistent AF	3 (9.7%)	5 (33%)	0.092
Comorbidities			
Hypertension	26 (84%)	12 (80%)	1.0
Diabetes mellitus	9 (29%)	3 (20%)	0.723
Heart failure	18 (58%)	9 (60%)	1.0
Coronary artery disease	4 (13%)	2 (13%)	1.0
Peripheral arterial disease	2 (6.5%)	1 (6.7%)	1.0
History of ischemic stroke	11 (36%)	9 (60%)	0.204
Chronic kidney disease	17 (55%)	9 (60%)	0.204
Chronic dialysis	2 (6.5%)	0	1.0
Embolisms			
Once	10 (32%)	8 (53%)	0.208
≧Twice	2 (6.5%)	3 (20%)	0.311
History of bleeding			
All bleeding	6 (19%)	6 (40%)	0.165
Major bleeding	2 (6.5%)	6 (40%)	0.010
Cerebral hemorrhage	2 (6.5%)	1 (6.7%)	1.0
Gastrointestinal bleeding	1 (3.2%)	1 (6.7%)	1.0
Risk scores			
CHADS_2_ score	3.3 ± 1.3	3.4 ± 0.9	0.744
CHA_2_DS_2_‐VASc score	4.9 ± 1.6	4.7 ± 1.0	0.603
HAS‐BLED score	3.3 ± 0.9	3.4 ± 0.5	0.707
History of surgery			
Open heart surgery	2 (6.5%)	1 (6.7%)	1.0
Prior TAVI	0	0	‐
PriorTEER	2 (6.5%)	0	1.0
Prior PPM	4 (13%)	1 (6.7%)	1.0
TTE			
LVEF, %	45.3 ± 4.5	46.5 ± 6.9	0.549
LVDd, mm	52.7 ± 9.2	51.5 ± 10.3	0.691
LAD, mm	42.8 ± 7.7	45.4 ± 5.2	0.194
Labo data			
eGFR	55 ± 24	51 ± 18	0.591
Hemoglobin (g/dL)	12.4 ± 1.9	12.6 ± 1.5	0.690
OAC at the first procedure			
VKA	6 (19%)	3 (20%)	1.0
DOAC	25 (81%)	12 (80%)	1.0
APT at the first procedure			
Aspirin	1 (3%)	1 (6.7%)	1.0
Clopidogrel	3 (9.7%)	1 (6.7%)	1.0
Other	0	1 (6.7%)	0.326
CA procedure			
Acute procedure success	31 (100%)	15 (100%)	‐
Radiofrequency catheter ablation	25 (81%)	14 (93%)	0.399
Balloon ablation	6 (19%)	1 (6.7%)	0.399
PVI	31 (100%)	15 (100%)	‐
PWI	8 (26%)	8 (53%)	0.10
LA substrate ablation	4 (13%)	7 (47%)	0.024
CTI	22 (71%)	11 (73%)	1.0
SVCI	5 (16%)	5 (33%)	0.257
CA procedure time	164 ± 65	205 ± 84	0.110
Complication related CA	0	0	‐
LAAC procedure			
Procedure success	30 (97%)	15 (100%)	1.0
WATCHMAN device	5 (16%)	3 (20%)	1.0
WATCHMAN FLX device	26 (84%)	12 (80%)	1.0
Max LAA ostium diameter	24.1 ± 3.3	25.5 ± 3.4	0.207
Device size	29.8 ± 3.8	31.8 ± 2.8	0.055
LAAC procedure time	44 ± 13	48 ± 20	0.438
Complication related LAAC	1 (3.2%)	0	1.0
Pericardial effusion	1 (3.2%)	0	1.0

*Note*: Categorical variables are presented as numbers and percentages, and continuous variables are presented as mean and standard deviation or median and interquartile range based on their distributions. Categorical variables were compared using chi‐squared test. Continuous variables were compared using one‐way analysis of variance or Kruskal–Wallis test based on their distributions. Heart failure was defined as a condition requiring intervention such as oral diuretics. Chronic kidney disease was diagnosed if there was persistent proteinuria or if the estimated glomerular filtration rate (eGFR) was <60 mL/min/1.73m^2^ for more than 3 months. The eGFR was calculated based on the equation reported by Japan Association of Chronic Kidney Disease Initiative [male: 194 × Scr − 1.094 × age−0.287, female: 194 × Scr − 1.094 × age−0.287 × 0.739]. History of major bleeding was diagnosed if the patient had a history of International Society on Thrombosis and Hemostasis major bleeding. LA substrate ablation was including such as low voltage ablation and complex fractionated electrogram ablation.

Abbreviations: APT, antiplatelet therapy; BMI, body mass index; CA, catheter ablation; CAD, coronary artery disease; CTI, cavo‐tricuspid isthmus isolation; DOAC, direct oral anticoagulant; LAAC, left atrial appendage (LAA) closure; LAD, left atrial diameter; LVDd, left ventricular diastolic diameter; LVEF, left ventricular ejection fraction; OAC, oral anticoagulant; PAD, peripheral arterial disease; PCI, percutaneous coronary intervention; PM, permanent pacemaker; PVI, pulmonary vein isolation; PWI, posterior wall isolation; SVCI, superior vena cava isolation; TAVI, transcatheter aortic valve implantation; TEER, transcatheter edge‐to‐edge repair; TTE, transthoracic echocardiography; VKA, vitamin K antagonist.

### 3.2 Procedural characteristics

Procedural characteristics were different in some aspects between the two groups. Regarding the CA procedure, the procedure time was longer in the LAAC first group than in the CA first group. Regarding the LAAC procedure, the device size was larger in the LAAC first group than in the CA first group.

### 3.3 Events from the first procedure to the second procedure

**FIGURE 2:**
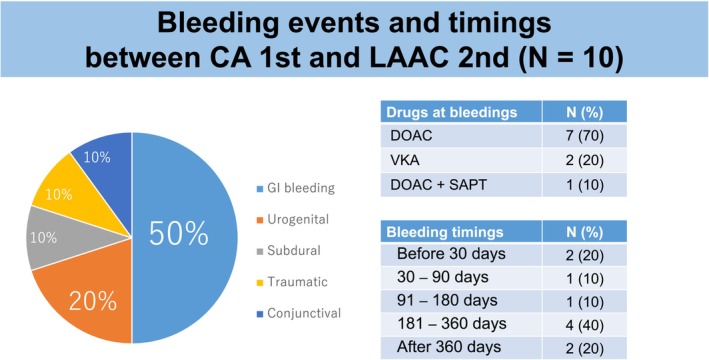
Bleeding events between the catheter ablation and percutaneous left atrial appendage closure procedures in the left atrial catheter ablation first group. (Left) Pie chart for the detailed types of bleeding events between the CA and LAAC procedures. (Right) Drugs being used at the time of the bleeding events. CA, catheter ablation; DOAC, direct oral anticoagulant; GI, gastrointestinal; LAAC, percutaneous left atrial appendage closure; SAPT, single antiplatelet therapy; VKA, vitamin K antagonist.

Events from the first procedure to the second procedure are shown in Table 2. In the CA first group, 10 patients experienced bleeding and 7 patients experienced major bleeding between the CA and LAAC procedures. Bleeding commonly occurred within 90 days and between 181 and 360 days. Gastrointestinal bleeding was the most common type of bleeding (Figure 2). All bleeding events were associated with oral anticoagulant therapy. There were seven patients on direct oral anticoagulants (DOACs), two on vitamin K antagonists (VKAs), and one on DOAC and single antiplatelet therapy. In the LAAC first group, no bleeding events were observed during the period from LAAC to CA.

**TABLE 2:** Results of cardiovascular events within the following period.

	Conventional group (*N* = 31)	LAAC first group (*N* = 15)	*p*‐value
Between 1st procedure to 2nd procedure			
Observation period	277 ± 197	212 ± 172	0.262
Events			
All clinically relevant bleedings	10 (32%)	0	0.019
Major bleedings	7 (23%)	0	0.078
Before 90 days	3/7	0	
90–180 days	1/7	0	
181–360 days	3/7	0	
After 360 days	0	0	
Ischemic Strokes	3 (10%)	2 (13%)	0.656
Nondisabling ischemic strokes	3 (10%)	2 (13%)	0.656
Disabling ischemic strokes	0	0	‐
Before 90 days	2/3	1/2	
90–180 days	1/3	0	
181–360 days	0	1/2	
After 360 days	0	0	
Heart failure hospitalization	3 (10%)	3 (20%)	0.375
Before 90 days	1/3	0	
90–180 days	2/3	0	
181–360 days	0	2/3	
After 360 days	0	1/3	
After both procedures			
Observation period	464 ± 277	420 ± 260	0.599
Events			
All clinically relevant bleedings	2 (6.5%)	0	1.0
Major bleedings	2 (6.5%)	0	1.0
Before 90 days	0	0	
90–180 days	1/2	0	
181–360 days	0	0	
After 360 days	1/2	0	
Ischemic Strokes	1 (3.2%)	0	1.0
Nondisabling ischemic strokes	1 (3.2%)	0	1.0
Disabling ischemic strokes	0	0	‐
After 360 days	1/1	0	
Heart failure hospitalization	5 (16%)	0	0.375
Before 90 days	2/5	0	
90–180 days	1/5	0	
181–360 days	2/5	0	
After 360 days	0	0	
All‐cause death	4 (13%)	0	0.288
Cardiovascular death	2 (6.5%)	0	1.0

*Note*: Categorical variables are presented as numbers and percentages, and continuous variables are presented as mean and standard deviation or median and interquartile range based on their distributions. Categorical variables were compared using chi‐squared test. Continuous variables were compared using one‐way analysis of variance or Kruskal–Wallis test based on their distributions. Strokes were classified as disabling if associated with an increase in the modified Rankin scale (mRS) score by at least 2 points at discharge.

Ischemic stroke occurred in three patients in the CA first group and in 2 patients in the LAAC first group, all of which were nondisabling ischemic strokes (Table S1).

HF hospitalization events were observed in three patients in the CA first group and three patients in the LAAC first group. In the CA first group, ischemic stroke and HF hospitalization events were more frequent within 180 days.

### 3.4 Indication for LAAC after CA

Indications for LAAC are shown in Table 3. The most common indication for LAAC after CA was prior major bleeding. Events led to considering LAAC indication are shown in Table S2.

**TABLE 3:** Indications for percutaneous left atrial appendage closure.

	Conventional group (*N* = 31)	LAAC first group (*N* = 15)
LAAC indication		
History of major bleeding	18 (58%)	6 (40%)
HAS‐BLED score ≥3 points	5 (16%)	7 (47%)
DAPT required ≥1 year	1 (3.2%)	0
Increased risk of fall	2 (6.5%)	0
Others	5 (16%)	2 (13%)

### 3.5 Indication for CA after LAAC or LAAC first

Indication for CA after LAAC or LAAC first is shown in Table 4. The most common indication for CA after LAAC was HF events, followed by palpitation events. The reason for performing LAAC first was a history of LAA thrombosis or LAA sludge along with, a high risk of bleeding. All patients with prior LAA thrombosis or LAA sludge had a history of major bleeding or were at high risk of bleeding (HAS‐BLED score ≥ 3 points).

**TABLE 4:** Reasons for ablation after percutaneous left atrial appendage closure or percutaneous left atrial appendage closure first.

	LAAC first group (*N* = 15)	LAAC first group in the early phase (*N* = 7)
Reason for ablation after LAAC	11 (73%)	3 (43%)
Heart failure event	4 (27%)	0
Palpitation event	3 (20%)	1 (14%)
BTS with symptoms	2 (13%)	2 (29%)
Paroxysmal AF change to persistent AF	2 (13%)	0
Reason for LAAC first	4 (27%)	4 (57%)
Prior LAA thrombosis	2 (13%)	2 (29%)
History of major bleeding	0	0
HAS‐BLED score ≥ 3 points	2 (13%)	2 (29%)
LAA sludge	2 (13%)	2 (29%)
History of major bleeding	2 (13%)	2 (29%)
HAS‐BLED score ≥ 3 points	0	0

*Note*: Categorical variables are presented as numbers and percentages.

Abbreviations: AF, atrial fibrillation; BTS, bradycardia–tachycardia syndrome; CA, catheter ablation; DAPT, dual antiplatelet therapy; LAA, left atrial appendage; LAAC, percutaneous left atrial appendage closure.

### 3.6 Events after both procedures

Events after both procedures are shown in Table 2. The mean observation periods for both procedures were 464 ± 277 days in the CA first group and 420 ± 260 days in the LAAC first group. As for procedure‐related adverse events, pericardial effusion was observed in one patient after LAAC in the CA first group. The patient underwent pericardiocentesis 140 days after LAAC. As for the cardiovascular adverse events after both procedures, no cardiovascular adverse events were observed in the LAAC first group. Major bleeding events were observed in two patients in the CA first group. An ischemic stroke event was observed in one patient in the CA first group, and it was a nondisabling ischemic stroke (Table S1). HF hospitalization events were observed in five patients in the CA first group. All‐cause death events were experienced in three patients in the CA first group, including cardiovascular death in two patients. Regarding these death events, one patient had HF after 280 days, one had coronavirus infection, and one had sudden cardiac death after 633 days.

### 3.7 Profile of the LAAC first procedure

One patient underwent cryoballoon ablation, and the other patients underwent RF ablation. PVI was performed in all CA procedures. In seven patients, CA was performed in the early phase within 180 days after LAAC, and in six patients, CARTOSOUND was used. Figure S2 shows the bipolar voltage on the device surface in the first session ablation cases that could be evaluated using Octaray and CARTOSOUND. The detailed evaluation of bipolar voltages at the device surface is shown in Table S3 and Figure S3. Patients with larger LAAC devices (35 mm) and larger low bipolar voltage area on the LA (cases 2 and 4) had delayed normalization of the bipolar voltage area on the LAAC device surface.

### 3.8 Imaging evaluation after both procedures

After completion of the CA procedure after LAAC, imaging evaluation (transesophageal echocardiography or contrast‐enhanced 64‐slice cardiac CT) was performed at 3 months after CA in 7 patients and at 12 months after CA in 4 patients. There were no device‐related adverse events (DRT, new PDL appearance, progressive increase in PDL, or device dislodgement) in the LAAC first group, including those in the early phase after LAAC (Table S4A).

After completion of the LAAC procedure after CA, imaging evaluation was performed at 3 months after CA in 22 patients and at 12 months after CA in 15 patients. There was no DRT in the CA first group, including those in the early phase after CA. However, there was 1 patient with progressive increase in PDL and 3 patients with new PDL appearance in the CA first group (Table S4B). In addition, there was 1 patient with progressive increase in PDL at 3 months and 2 patients with new PDL appearance at 12 months in patients undergoing LAAC in the early phase after CA.

### 3.9 Antithrombotic regimen

**FIGURE 3:**
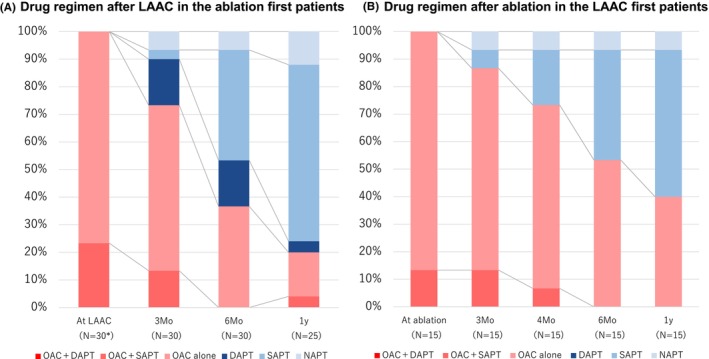
Oral antithrombotic drug regimen. (A) Oral antithrombotic drug regimen after left atrial appendage closure in the catheter ablation first group. *Excluding 1 left atrial appendage closure failure patient. (B) Oral antithrombotic drug regimen after ablation in the left atrial appendage closure first group. DAPT, dual antiplatelet therapy; LAAC, percutaneous left atrial appendage closure; NAPT, no antiplatelet therapy; OAC, oral anticoagulant; SAPT, single antiplatelet therapy.

Antithrombotic drug regimens after LAAC in the CA first patients and those after CA in the LAAC first patients were shown in Figure 3. All patients undergoing CA after LAAC received oral anticoagulants. After completion of the CA procedure after LAAC, oral anticoagulants were discontinued in nine patients 1 year after ablation. The antithrombotic drug regimen for each patient's background was shown in Figure S4 (with or without a history of major bleeding), and Figure S5 (with or without a history of ischemic stroke). Oral anticoagulants were discontinued at 1 year in all patients who had not experienced ischemic stroke.

## 4 DISCUSSION

The present study had some important findings. First, there were no difference in procedure‐related adverse events and cardiovascular adverse events after both procedures. Moreover, after completing both procedures, there was no device‐related adverse event (DRT, new PDL appearance, progressive increase in PDL, or device dislodgement) in LAAC first group, whereas there were 4 device‐related adverse events in the CA first group. Second, the most common reason for CA after LAAC was HF events, and the most common reason for performing LAAC first was the patients with a history of LAA thrombosis or LAA sludge, who had a history of major bleeding or were at high risk of bleeding (HAS‐BLED score ≥ 3 points). Third, we observed device surface voltage for each patient in the LAC first patients.

### 4.1 Ablation after LAAC device implantation and LAAC device‐related adverse events

A progressive increase in PDL has been reported with ablation treatment after LAAC.^20^ In this study, transesophageal echocardiography was performed in five patients at 3 months after CA, and four patients at 12 months after the CA procedure post‐LAAC; however, a progressive increase in PDL was not observed postoperatively. There was one case of minor PDL (2 mm) before the CA procedure, but there was no change in PDL (2 mm) in transesophageal echocardiography 9 months after CA. DRT has been reported to occur in 12.5% of patients after left atrial RF ablation.^21^ In this study, postoperative imaging evaluation (transesophageal echocardiography or contrast‐enhanced 64‐slice cardiac CT) at 3 months after CA was performed in 7 patients, at 12 months after CA was performed in 4 patients; no DRT was observed in any patient. This study was performed with WATCHMAN 2.5 or WATCHMAN FLX device only. If an Amplatzer Amulet device is used,^22^ the device disk may overlap the left pulmonary vein ridge and interfere with ablation of the ridge, including PVI.

### 4.2 Simultaneous procedure and LAAC first strategy

It has been previously reported that the average simultaneous procedure time for LAAC and CA was 177 min. In present study, the LAAC first group included more long‐standing persistent AF, so the procedure time was longer in the LAAC first group. The advantages of simultaneous procedure were that it could be completed in only one session and that the total procedure time was shorter than separate procedures, whereas, the disadvantage of simultaneous procedure was that the major adverse cardiovascular events numerically increased due to simultaneous procedures performed in one session,^18^ and that seems to be expected in the improvement of the LAAC device. Actually, it has been previously reported that transition to WATCHMAN FLX reduces perioperative complications, so this may be resolved in the future even Japan.^11^

In previous reports of simultaneous procedures combining CA and LAAC, the simultaneous procedure (CA followed by LAAC) may result in increased PDL and decreased device compression rate over time due to left atrial ridge edema associated PVI.^26^, ^27^ In this study, patients with a shorter time to LAAC after ablation (in the early phase ≤180 days) had a numerically slightly higher new PDL appearance and progressive increase in PDL. This result is similar to previous studies,^26^, ^27^ and the shorter the period after CA, the PDL after LAAC is more likely to be affected by LAA edema. Therefore, when we perform LAAC after PVI, it may be better to wait at least 180 days, considering the possibility of PDL due to LAA edema.

In previous reports, 15.6%–24.8% of patients who had undergone LAAC had HF, but LAAC was safely performed without increasing heart‐related complications.^28^, ^29^ In this study, HF hospitalization after LAAC first occurred in the late phase after 180 days of LAAC. Therefore if AF ablation is necessary to resolve HF in the late phase after 180 days of LAAC, it is considered safe, and feasible.^23^ Although there were many patients with a history of major bleeding in LAAC's first strategy group, no bleeding events including major bleeding occurred during the observation period. Therefore, an LAAC first strategy may be considered in patients with a relatively low risk of HF, and a high bleeding risk such as a history of major bleeding.

### 4.3 LAAC device endothelialization and bipolar voltage

Robust endothelialization of the WATCHMAN 2.5 or FLX device at the atrial surface by 28–90 days has been reported in a canine model,^30^ suggesting tissue ingrowth as a source of chronic stability, but this is known to be delayed in humans. Moreover, the variability in organized neoendocardial coverage over the device was due to placement within the appendage.^31^ Not only changes over time but also device size, type, and position, pressure contact with the LAA wall, and rhythm affect endothelialization.^30^, ^31^, ^32^ In addition, it has been reported that the bipolar voltage may be able to estimate the degree of endothelialization of the LAAC device surface.^32^ In this study, patients with large WATCHMAN FLX devices (35 mm) and larger low bipolar voltage area on the LA had a small normal bipolar voltage area on the LAAC device surface. As a note, patients with large devices such as 35 mm and larger low bipolar voltage area on the LA may show a small normal voltage zone due to less far‐field potential, not due to delayed endothelialization of the LAAC device. However, this requires further investigation, including assessment of the above factors.

### 4.4 Study limitation

The present study had several limitations. First, this was an observational and nonrandomized study performed at a single center, and the number of cases was very small. In addition, the number of cases who underwent CA in the early phase after LAAC first was more very small. Second, the preprocedural bleeding events between CA first and LAAC second was may have influenced by selection bias for LAAC indication due to bleeding events after CA. Third, the decisions, including CA strategies and anticoagulation strategies, were left to the discretion of the attending physician. Fourth, LAAC devices such as Amplatzer Amulet other than WATCHMAN devices were not considered. Fifth, only a few patients had undergone postprocedural transesophageal echocardiography to confirm for secondary leakage of the LAAC device. This study only provided initial small observational results from a single center and formulated a hypothesis. Further studies with a large number of cases and randomized trials are required.

## 5 CONCLUSION

Based on our small single‐center experience, left atrial CA therapy in the presence of an LAAC device implanted including the early phase was safe and feasible. The LAAC first strategy may be considered in patients with (1) prior major bleeding and/or high bleeding risk (HAS‐BLED score ≥3 points), (2) prior LAA thrombosis or a high risk of embolism including LAA sludge, and (3) a low risk of HF. When we perform LAAC after PVI, it may be better to wait at least 180 days, considering the possibility of PDL due to LAA edema.

## 6 FUTURE CONSIDERATIONS

In countries and institutions where simultaneous application of LAAC and CA is not possible, CA therapy is expected to increase after LAAC as LAAC devices become more and more popular. According to the ICE guidelines, this strategy is considered safe and feasible. Proficiency in using ICE is required. In addition, depending on the situation, the LAAC first strategy may be considered in some patients, but this requires further examination with a larger number of cases.

## FUNDING INFORMATION

This research received no specific grant from any funding agency.

## CONFLICT OF INTEREST STATEMENT

Dr. Kubo is a clinical proctor for Boston Scientific. Dr. Kubo received honorarium from Boston Scientific. All other authors declare no conflict of interest.

## ETHICS APPROVAL STATEMENT

This study was conducted with the approval of the Ethics Committee of Kurashiki Central Hospital (approval no. 4133).

## PATIENT CONSENT STATEMENT

Written informed consent from each patient was waived, because we used clinical information obtained in routine clinical practice and none of the patients refused to participate in the study when contacted for follow‐up. This method is concordant with the guidelines for epidemiological studies issued by the Ministry of Health, Labor, and Welfare in Japan.

## Supporting information


Data S1.

## Data Availability

The data that support the findings of this study are available from the corresponding author upon reasonable request.
