# Supplementary material for: Safety and feasibility of atrial fibrillation ablation after left atrial appendage closure: A single‐center experience of the left atrial appendage closure first strategy
Source: J Arrhythm. 2024 May 23;40(4):879–90. doi: 10.1002/joa3.13073 (PMC11317688; doi:10.1002/joa3.13073)
Supplement: Supplementary file 1 — Data S1. [file JOA3-40-879-s001.docx]

**Supplementary File**

**Supplementary Figure**

**Supplementary Figure 1.** Strategy for left pulmonary vein ridge ablation

**Supplementary Figure 1-(a)**

**
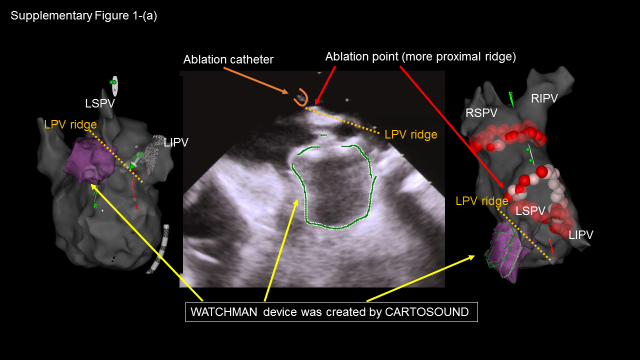
**

Supplementary Figure 1-(a).

Our ridge ablation strategy to achieve pulmonary vein isolation without interfering with the WATCHMAN device is as follows: first, we tried to ablate the ridge from the pulmonary vein side more proximal to the ridge using intracardiac echocardiography.

**Supplementary Figure 1-(b)**


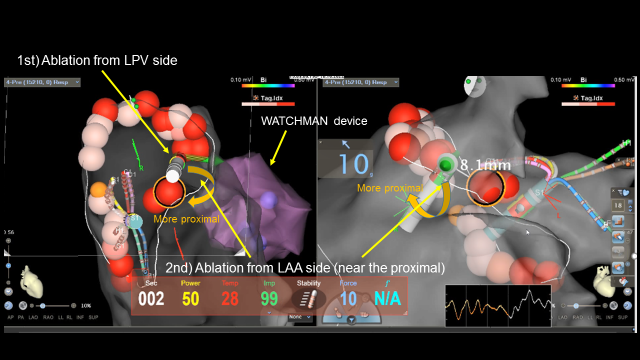


Supplementary Figure 1-(b).

Our ridge ablation strategy for achieving PVI to avoid interfering with the WATCHMAN device is as follows: First, we tried to ablate the ridge from the PV side more proximal ridge using ICE. If PVI was not achieved, we then tried ablate the ridge from the LAA side near the proximal side to avoid interference with the LAAC device.

LPV, left pulmonary vein; PVI, pulmonary vein isolation; ICE, intracardiac echocardiography; LAA, left atrial appendage.

**Supplementary Figure 2.** Patient characteristics and surface voltage of the left atrial appendage closure device at ablation in the percutaneous left atrial appendage closure first group


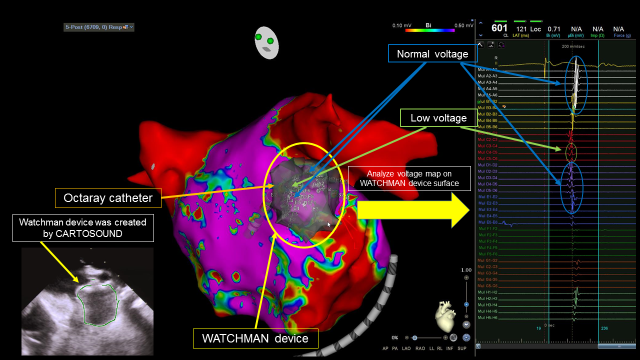


Supplementary Figure 2.

First, an LAAC device was created by CARTOSOUND. Second, bipolar voltage at the LAAC device surface was measured using an Octaray mapping catheter. The observed cases consisted only of the first session ablation cases with CARTOSOUND.

LAAC, percutaneous left atrial appendage closure; LVZA, low voltage zone area; NVZA, normal voltage zone area.

**Supplementary Figure 3.** The surface voltage map of the left atrial appendage closure device and the left atrial voltage map after ablation

**Supplementary Figure 3-(a)**


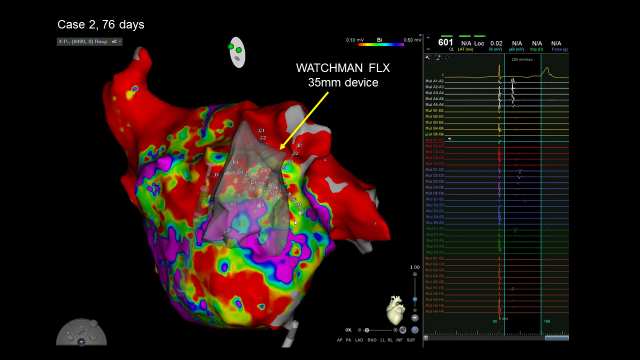


Supplementary Figure 3-(a).

The surface voltage map of the left atrial appendage closure device and the left atrial voltage map after ablation in case 2.

**Supplementary Figure 3-(b)**


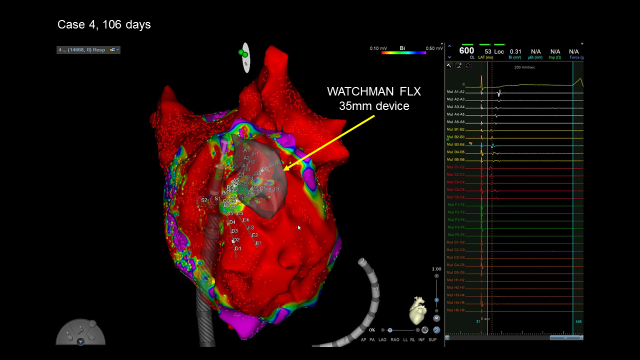


Supplementary Figure 3-(b).

The surface voltage map of the left atrial appendage closure device and the left atrial voltage map after ablation in case 4.

**Supplementary Figure 3-(c)**


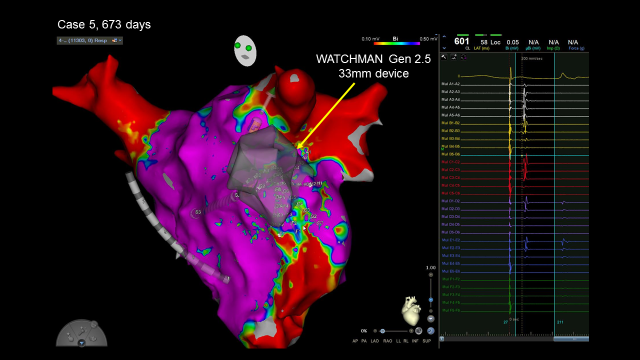


Supplementary Figure 3-(c).

The surface voltage map of the left atrial appendage closure device and the left atrial voltage map after ablation in case 5.

**Supplementary Figure 4.** Oral antithrombotic drug regimen after ablation in the percutaneous left atrial appendage closure first group

**
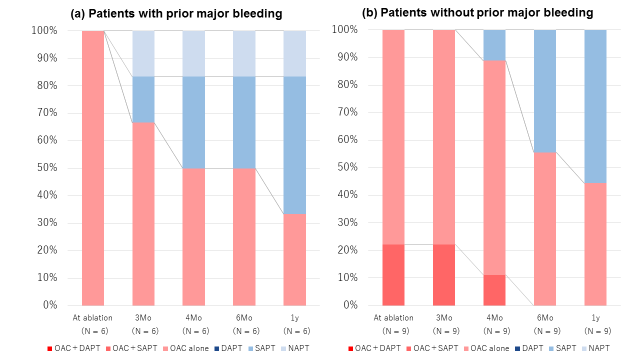
**

**Supplementary Figure 4.**

(a) Patients with a history of major bleeding, (b) Patients without a history of major bleeding. Abbreviations as in Figure 3.

**Supplementary Figure 5.** Oral antithrombotic drug regimen after ablation in the percutaneous left atrial appendage closure first group

**
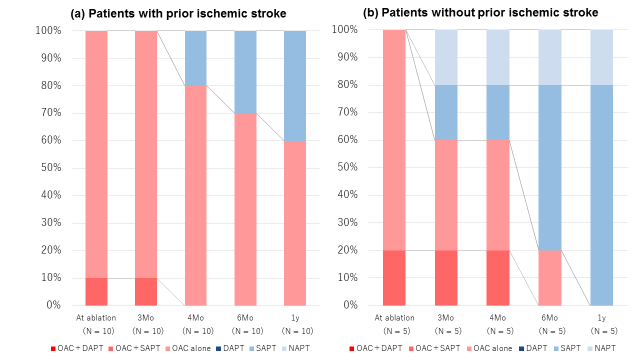
**

**Supplementary Figure 5.**

(a) Patients with a history of ischemic stroke, (b) Patients without a history of ischemic stroke.

History of ischemic stroke includes those occurring between the LAAC and ablation.

Abbreviations as in Figure 3.

**Supplementary Table**

**Supplementary Table 1:** Detailed ischemic stroke events including the modified Rankin scale

|  | **mRS**  **before admission** | **mRS**  **on admission** | **mRS**  **at discharge** |
| --- | --- | --- | --- |
| **Between 1st and 2nd procedures** |  |  |  |
| Ischemic strokes (CA first group; n = 3) |  |  |  |
| Case 1 (42 days, nondisabling stroke) | 0 points | 0 points | 0 points |
| Case 2 (46 days, nondisabling stroke) | 4 points | 4 points | 4 points |
| Case 3 (154 days, nondisabling stroke) | 0 points | 0 points | 0 points |
| Ischemic strokes (LAAC first group; n = 2) |  |  |  |
| Case 1 (3 days, nondisabling stroke) | 0 points | 0 points | 1 point |
| Case 2 (237 days, nondisabling stroke) | 2 points | 2 points | 2 points |
| **After both procedures** |  |  |  |
| Ischemic strokes (CA first group; n = 1) |  |  |  |
| Case 1 (431 days, nondisabling stroke) | 0 points | 0 points | 0 points |
| Ischemic strokes (LAAC first group; n = 0) |  |  |  |

Strokes were classified as disabling if associated with an increase of at least 2 points in the modified Rankin scale (mRS) score at discharge.

**Supplementary Table 2:** Events led to considering percutaneous left atrial appendage closure treatment indication

|  | **Conventional group**  **(N = 31)** |
| --- | --- |
| **Events led to considering LAAC indication** |  |
| Bleeding event | 10 (32%) |
| Embolic event | 3 (10%) |
| Prior bleeding event | 5 (16%) |
| Prior embolic event | 7 (23%) |
| Others | 6 (19%) |

**Supplementary Table 3:** Detailed patient characteristics and surface voltage of the left atrial appendage closure device at ablation in the percutaneous left atrial appendage closure first group

|  | **Case 1** | **Case 2** | **Case 3** | **Case 4** | **Case 5** |
| --- | --- | --- | --- | --- | --- |
| Age, years | 74 | 77 | 62 | 74 | 69 |
| Sex | Female | Male | Female | Female | Male |
| CHADS_2_ score | 3 | 4 | 3 | 5 | 4 |
| CHA₂DS₂-VASc score | 5 | 5 | 3 | 7 | 4 |
| HAS-BLED score | 4 | 3 | 3 | 3 | 4 |
| AF type | PAF | PEF | LSAF | PEF | PEF |
| LVEF | 57% | 60% | 35% | 43% | 60% |
| LAD | 40 mm | 48 mm | 52 mm | 48 mm | 39 mm |
| LAAC device size | 27 mm | 35 mm | 27 mm | 35 mm | 30 mm |
| WATCHMAN type | FLX | FLX | FLX | FLX | Gen 2.5 |
| After LAAC, days | 39 | 76 | 103 | 106 | 673 |
| LVZA < 0.5 mV on the LA | 10% | 78% | 16% | 86% | 2% |
| NVZA ≥ 0.5 mV on the LA | 90% | 22% | 84% | 14% | 98% |
| LVZA < 0.5 mV on the LAAC device | 44% | 85% | 26% | 99% | 15% |
| NVZA ≥ 0.5 mV on the LAAC device | 56% | 15% | 74% | 1% | 85% |

LAAC, percutaneous left atrial appendage closure; AF, atrial fibrillation; PAF, paroxysmal AF; PEF, persistent AF; LSAF, long standing persistent AF; LVEF, left ventricular ejection fraction; LAD, left atrial diameter; LAA, left atrial appendage; LVZA, low voltage zone area; NVZA, normal voltage zone area.

**Supplementary Table 4-(a):** Image evaluation after ablation after percutaneous left atrial appendage closure first

|  | **LAAC first group**  **(N = 15)** | **LAAC first group**  **in the early phase**  **(N = 7)** | **LAAC first group**  **in the late phase**  **(N = 8)** |
| --- | --- | --- | --- |
| **TEE at LAAC procedure** |  |  |  |
| Complete seal | 14 (93%) | 7 (100%) | 7 (88%) |
| PDL | 1 (6.7%) | 0 | 1 (13%) |
| < 3 mm | 1 (6.7%) | 0 | 1 (13%) |
| ≥ 3 mm | 0 | 0 | 0/8 |
| **Imaging 3 months after ablation** |  |  |  |
| CCT at 3 months after ablation | 2 (13%) | 1 (14%) | 1 (13%) |
| TEE at 3 months after ablation | 5 (33%) | 2 (29%) | 3 (38%) |
| DRT at 3 months after ablation | 0/7 | 0/3 | 0/4 |
| Complete seal by TEE | 5/5 (100%) | 2/2 (100%) | 3/3 (100%) |
| PDL by TEE | 0/5 | 0/2 | 0/3 |
| **Imaging 12 months after ablation** |  |  |  |
| CCT at 12 months after ablation | 0 | 0 | 0 |
| TEE at 12 months after ablation | 4 (27%) | 2 (29%) | 2 (25%) |
| DRT at 12 months after ablation | 0/4 | 0/2 | 0/2 |
| Complete seal by TEE | 3/4 (75%) | 1/2 (50%) | 2/2 (100%) |
| PDL by TEE | 1/4 (25%) | 1/2 (50%) | 0/2 |
| New PDL appearance after ablation | 0/4 | 0/2 | 0/2 |
| Progressive increase in PDL after ablation | 0/4 | 0/2 | 0/2 |
| < 3 mm | 1/4 (25%) | 1/2 (50%) | 0/2 |
| ≥ 3 mm | 0/4 | 0/2 | 0/2 |

**Supplementary Table 4-(b):** Image evaluation after left atrial appendage closure after catheter ablation first

|  | **Conventional group**  **(N = 30)*** | **Conventional group**  **in the early phase**  **(N = 14)** | **Conventional group**  **in the late phase**  **(N = 16)** |
| --- | --- | --- | --- |
| **TEE at LAAC procedure** |  |  |  |
| Complete seal | 28 (93%) | 13 (93%) | 15 (94%) |
| PDL | 2 (6.7%) | 1 (7.1%) | 1 (6.3%) |
| < 3 mm | 2 (6.7%) | 1 (7.1%) | 1 (6.3%) |
| ≥ 3 mm | 0 | 0 | 0 |
| **Imaging 3 months after LAAC** |  |  |  |
| CCT at 3 months after LAAC | 4 (13%) | 1 (7.1%) | 3 (19%) |
| TEE at 3 months after LAAC | 22 (73%) | 11 (64%) | 11 (69%) |
| DRT at 3 months after LAAC | 0/26 | 0/12 | 0/14 |
| Complete seal by TEE | 20/22 (91%) | 10/11(91%) | 10/11 (91%) |
| PDL by TEE | 2/22 (9.1%) | 1/11 (9.1%) | 1/11 (9.1%) |
| New PDL appearance | 1/22 (4.5%) | 0/11 | 1/11 (9.1%) |
| Progressive increase in PDL | 1/22 (4.5%) | 1/11 (9.1%) | 0/11 |
| < 3 mm | 1/22 (4.5%) | 0/11 | 1/11 (9.1%) |
| 3 – 5mm | 1/22 (4.5%) | 1/11 (9.1%) | 0/11 |
| **Imaging 12 months after LAAC** |  |  |  |
| CCT at 12 months after LAAC | 6 (20%) | 4 (29%) | 2 (13%) |
| TEE at 12 months after LAAC | 15 (50%) | 7 (50%) | 8 (50%) |
| DRT at 12 months after LAAC | 0/21 | 0/11 | 0/10 |
| Complete seal by TEE | 11/15 (73%) | 4/7 (57%) | 7/8 (88%) |
| PDL by TEE | 4/15 (27%) | 3/7 (43%) | 1/8 (13%) |
| New PDL appearance | 2/15 (13%) | 2/7 (29%) | 0/8 |
| Progressive increase in PDL | 0/15 | 0/7 | 0/8 |
| < 3 mm | 4/15 (27%) | 3/7 (43%) | 1/8 (13%) |
| ≥ 3 mm | 0/15 | 0/7 | 0/8 |

*Excluding one LAAC failure patient.

Categorical variables are presented as numbers and percentages. Image evaluation of the allowable range for the period was 60 days before and after. DRT was evaluated by CCT or TEE.

LAAC, percutaneous left atrial appendage closure; TEE, transesophageal echocardiography; CCT, contrast 64-slice cardiac computed tomography; DRT, device-related thrombosis; PDL, peri-device leakage.
